# Magnetic resonance spectroscopy investigation in the right human hippocampus following spinal cord injury

**DOI:** 10.3389/fneur.2023.1120227

**Published:** 2023-05-12

**Authors:** Dario Pfyffer, Sandra Zimmermann, Kadir Şimşek, Roland Kreis, Patrick Freund, Maryam Seif

**Affiliations:** ^1^Spinal Cord Injury Center, Balgrist University Hospital, University of Zürich, Zürich, Switzerland; ^2^Department of Anesthesiology, Perioperative and Pain Medicine, Stanford University School of Medicine, Palo Alto, CA, United States; ^3^Magnetic Resonance Methodology, Institute of Diagnostic and Interventional Neuroradiology, University of Bern, Bern, Switzerland; ^4^Translational Imaging Center (TIC), Swiss Institute for Translational and Entrepreneurial Medicine, Bern, Switzerland; ^5^Graduate School for Cellular and Biomedical Sciences, University of Bern, Bern, Switzerland; ^6^Department of Neurophysics, Max Planck Institute for Human Cognitive and Brain Sciences, Leipzig, Germany

**Keywords:** SCI – spinal cord injury, cognitive impairment, MRS – 1H nuclear magnetic resonance spectroscopy, hippocampus, sLASER sequence

## Abstract

**Objective:**

Preclinical studies have shown that cognitive impairments following spinal cord injury (SCI), such as impaired spatial memory, are linked to inflammation, neurodegeneration, and reduced neurogenesis in the right hippocampus. This cross-sectional study aims to characterize metabolic and macrostructural changes in the right hippocampus and their association to cognitive function in traumatic SCI patients.

**Methods:**

Within this cross-sectional study, cognitive function was assessed in 28 chronic traumatic SCI patients and 18 age-, sex-, and education-matched healthy controls by a visuospatial and verbal memory test. A magnetic resonance spectroscopy (MRS) and structural MRI protocol was performed in the right hippocampus of both groups to quantify metabolic concentrations and hippocampal volume, respectively. Group comparisons investigated changes between SCI patients and healthy controls and correlation analyses investigated their relationship to memory performance.

**Results:**

Memory performance was similar in SCI patients and healthy controls. The quality of the recorded MR spectra was excellent in comparison to the best-practice reports for the hippocampus. Metabolite concentrations and volume of the hippocampus measured based on MRS and MRI were not different between two groups. Memory performance in SCI patients and healthy controls was not correlated with metabolic or structural measures.

**Conclusion:**

This study suggests that the hippocampus may not be pathologically affected at a functional, metabolic, and macrostructural level in chronic SCI. This points toward the absence of significant and clinically relevant trauma-induced neurodegeneration in the hippocampus.

## Introduction

Spinal cord injury (SCI) is a debilitating event that results in immediate and potentially permanent sensorimotor dysfunctions (1). While SCI has been associated with an increased risk of cognitive decline, including attention deficits, slow processing speed, social communication impairments, and learning and memory dysfunctions (2–5), other studies did not reveal cognitive dysfunctions following SCI (6). A higher risk to develop dementia has been reported in SCI patients (7) and raises the question whether trauma-induced neurodegenerative processes can affect the limbic system and represent a correlate of cognitive impairments, such as memory deficits.

The hippocampus is particularly susceptible to adverse neurological conditions such as Alzheimer’s disease (AD) (8, 9), schizophrenia (10), and the aging process. (8, 9). While the verbal memory is primarily encoded in the bilateral heads of the hippocampi (11), the right hippocampus is involved in the formation of visuospatial memory (12) and is vulnerable to metabolic and cytotoxic conditions (e.g., ischemia, neuroinflammation, and neurodegeneration) (8). Preclinical evidence showed a link between impaired spatial memory and chronic hippocampal inflammation, microglial activation, neurodegeneration, and reduced neurogenesis in experimental SCI models (13, 14). Although these processes have been reported to be related to hippocampal damage and memory impairments in human SCI (15), concrete evidence of such is still missing.

Magnetic resonance spectroscopy (MRS) and imaging (MRI) are promising non-invasive techniques to quantitatively explore secondary neurodegenerative processes in the injured central nervous system (16). The aim of this study was therefore to quantify metabolic and volumetric changes in the right human hippocampus and their potential relation to cognitive deficits in chronic SCI patients by means of MRS, structural MRI, and spatial memory tests. We hypothesized that SCI leads to remote structural and molecular changes in the right hippocampus that are associated with visuospatial memory performance.

## Methods

### Standard protocol approvals and patient consents

The study protocol was designed in accordance with the Declaration of Helsinki and approved by the local Ethics Committee (EK-2018-00937). Informed written consent was obtained from each participant before study enrolment.

### Participants

Twenty-eight patients with chronic traumatic SCI (2 female) and 18 age- and education-matched healthy controls (3 female) were recruited between March 2021 and July 2021 at the Spinal Cord Injury Center, Balgrist University Hospital, for this cross-sectional study. During the prospective recruitment process of the study, we took steps to ensure a comparable proportional distribution of age and education between both groups. This was done to minimize any potential confounding variables that could affect the study’s results and to increase the validity of our findings. The number of subjects was defined based on our previous study on SCI (17). Inclusion criteria for SCI patients were: chronic stage of SCI (more than 6 months post-injury), no additional diagnosed neurological or psychological disorders, and no MRI contraindications. Inclusion criteria for healthy controls were no neurological, cognitive, or psychological disorders, and no MRI contraindications. To guarantee that participants could comprehend the testing materials and instructions as well as to provide accurate responses in German, all study participants were required to be fluent in the language, including proficiency in speaking, reading, and writing. Demographics and injury characteristics of SCI patients are summarized in Table 1. During the study appointment, all subjects were asked about their highest level of education. Graduating from mandatory school, including elementary school or secondary school, was defined as category [I] and graduating from high school or university was ascribed to category [II]. Data on behavioral visuospatial and verbal memory performance, MRS measurements of the right hippocampus, and hippocampal volumetric MRI measurements were obtained from all study participants.

**Table 1:** Demographics and neurological information of spinal cord injury (SCI) patients.

Subject ID	Sex	Age (y)	Age at injury (y)	Time since injury (y)	NLI	AIS
1	Male	66	58	8	C3	A
2	Male	34	28	6	C4	A
3	Male	38	31	7	C4	A
4	Male	42	22	20	C7	A
5	Male	58	21	37	C7	A
6	Male	58	43	15	T1	A
7	Male	52	15	37	T4	A
8	Male	50	27	23	T5	A
9	Male	55	24	31	T6	A
10	Male	56	33	23	T7	A
11	Male	57	46	11	T11	A
12	Male	34	29	5	T12	A
13	Female	37	28	9	C4	B
14	Male	45	36	9	C7	B
15	Male	59	57	2	T2	B
16	Male	52	19	33	T4	B
17	Female	37	32	5	C5	C
18	Male	60	25	35	C6	C
19	Male	37	15	22	C7	C
20	Male	31	17	14	C7	C
21	Male	53	40	13	C2	D
22	Male	66	50	16	C4	D
23	Male	68	60	8	C4	D
24	Male	48	30	18	C5	D
25	Male	30	20	10	C5	D
26	Male	62	50	12	C5	D
27	Male	31	27	4	C7	D
28	Male	22	20	2	T12	D

NLI, neurological level of injury; AIS, American Spinal Injury Association Impairment Scale.

### Behavioral testing of visuospatial and verbal memory function

To examine hippocampus-related cognitive function, the visuospatial and verbal working memory functions were assessed using the test *‘Verbaler und Visueller Merkfähigkeitstest’* (VVM) (18). The VVM is a widely used (19–22) and standardized test assessing memory function in German language and includes two tasks as follows: the first task is called ‘Map’ which assesses the visuospatial memory function, and the second task is called ‘Text’ which examines the verbal memory function. For the ‘Map’ task, the SCI and healthy controls were asked to memorize a specific path through a city map within 2 min time. Immediately thereafter, which was time-point 1 (t1), participants were asked to recall the path with drawing it on the same city map without the path within 2 min. Every correctly crossed intersection was scored with one point and a maximum number of 31 points were achieved. For the ‘Text’ task, participants were asked to read and memorize a text including names, numbers, and other facts during 2 min. They had to answer questions related to the text within 4 min immediately after memorizing the text details (t1). Every correct answer was scored with one point and a maximum number of 24 points were scored. The recalling part of both tasks was repeated after the scans– approximately taking 90 min – and represented intermediate memory function at time-point 2 (t2) (18). SCI patients without or with very impaired hand function were assisted filling out the tests to counteract the limited time factor.

### Mini-mental state examination

We performed the Folstein Mini-Mental State Examination (MMSE) (23) with our study participants to explore potential cognitive deficits and to rule out participants with signs of dementia. We used a validated telephone version of the MMSE as part of the Adult Lifestyles and Function Interview (ALFI-MMSE) (24) to ensure the highest possible participation rate despite the limited availability of study participants. Of 28 SCI patients and 18 healthy controls, we were able to contact 22 patients and all healthy controls for the follow-up telephone interview. This version of the MMSE has a maximum score of 22 points, consisting of the subscale’s orientation to time, orientation to place, registration, attention, and recall. Study participants were categorized into without dementia, with mild dementia, with moderate dementia, or severe dementia, based on their scores.

### MRI and MRS acquisition

MRS and MRI measurements were performed on a 3 T MR scanner (Prisma, Siemens Healthcare, Erlangen, Germany) with a 64-channel receive head and neck radio frequency (RF) coil. The protocol consisted of structural T1-weighted (T1-w) and T2-weighted (T2-w) MRI sequences of the whole brain, B_0_ shimming, and MRS measurements in the right hippocampus and a reference region within the posterior parietal lobe (total acquisition time ≈60 min). The participants were scanned in a supine, head-first position and foam pads were used to minimize head motion in the RF coil.

As anatomical reference images, three sagittal, transversal, and coronal T2-weighted MRI scans based on a Turbo Spin Echo sequence were performed on the brain for subsequent MRS voxel placement on the hippocampus (echo time (TE) = 96 ms, echo spacing = 11 ms, repetition time (TR) = 5,000 ms, flip angle = 150°, in-plane resolution 0.5 × 0.5mm^2^, echo train length = 11). The T2-w MRI measurements were recorded separately for all three directions (sagittal, transversal, and coronal), first covering the whole brain (slice thickness of 4 mm) and then focally with better slice resolution (1.6 mm) to delineate the hippocampus anatomy in more detail and increase the accuracy for placing the MRS voxel. To accelerate the T2-w scanning, we used GRAPPA mode with acceleration factor of 2.

B_0_ shimming was performed using FASTESTMAP (25) prior to MRS measurement in the right hippocampus and posterior parietal lobe of the brain. The posterior parietal lobe served as an internal reference region since it is less affected by secondary neurodegeneration after SCI. Single-voxel MRS data were collected from a 25 × 12 × 8 mm^3^ (2.4 mL) voxel positioned in the right hippocampus and from a 20 × 14 × 22 mm^3^ (6.2 mL) voxel positioned in the parietal lobe. The MRS measurements were performed using a custom-made semi localization by adiabatic selective refocusing (sLASER) sequence (26) combined with metabolite cycling (MC) (27) [TE = 35 ms, TR = 2,500 ms, 1.024 s acquisition time, (only 0.512 s used)]. In total, 256 individually stored acquisitions were recorded across two blocks of 128 scans to reduce potential exclusion of whole datasets. Additionally, unsuppressed water signals with differing echo times (TEs = 35, 1,000, 50, 400, 200, 75, 100, and 140 ms, TR = 6,000 ms) were obtained to determine the parenchymal vs. cerebrospinal fluid (CSF) water signal for metabolite quantification. Outer volume suppression slabs were used to further prevent signal contamination from outer volume signals. Using sagittal, transversal, and coronal T2-w images, the volume of interest (VOI) was aligned parallel to the longitudinal axis of the right hippocampus and placed in a way such that it covered the spatial memory-relevant body and tail but not the head, which had been shown to be bilaterally associated with episodic memory (11) and verbal memory (28). T1-w magnetization-prepared rapid acquisition with gradient echo (MPRAGE) whole brain and upper cervical spinal cord images were acquired (TE = 2.32 ms, TR = 2,300 ms, flip angle = 8°, in-plane resolution 0.9 × 0.9mm^2^, slice thickness = 0.9 mm) to explore injury-induced macrostructural changes within the hippocampus in SCI patients using voxel-based morphometry (VBM) (29).

### MRI and MRS post-processing

Phase, frequency, and eddy-current correction as well as removal of motion-related artifacts were performed on the metabolite-cycled, non-water-suppressed MRS acquisitions using a motion compensation (MoCom) scheme in MATLAB 2020b (MathWorks, Natick, MA) (27). Spectra were quantified by linear combination model fitting using the fitting tool for arrays of interrelated datasets (FitAID) (30). The model basis set included simulated spectra of the following metabolites: aspartate, creatine (Cr), γ-aminobutyric acid (GABA), glucose, glutamate (Glu), glutamine (Gln), glutathione, glycine, glycerophosphorylcholine (GPC), lactate, myo-inositol (mI), N-acetylaspartate (NAA), N-acetylaspartylglutamate (NAAG), phosphocreatine (PCr), phosphorylcholine (PCho), phosphorylethanolamine, scyllo-inositol, and taurine. A macromolecular background (MMBG) signal was included in the basis set. It had been created based on a cohort average spectrum of the first 20 subjects by modeling an overall MMBG as a set of equally spaced Voigt lines (5 Hz spacing, 14 Hz Lorentz width, 10.6 Hz Gauss width) in addition to the metabolites’ basis spectra. Results are presented for total N-acetylaspartate (tNAA = NAA + NAAG), mI, choline-containing compounds (tCho = GPC + PCho), total creatine (tCr = Cr + PCr), and combined glutamate and glutamine (Glx = Glu + Gln) as these are relevant biochemicals that are known to be most reliably and reproducibly quantified in hippocampal MRS at 3 T (31, 32). The Cramér-Rao lower bound (CRLB) (33, 34) was calculated for each metabolite estimated. It represents the minimal error of fitting and is often used as an indicator of the data quality (33, 34) because it is affected by signal-to-noise, linewidth and model-based uncertainties. Here, the CRLBs are used in addition to a pure linewidth criterion as a way to filter out spectra of unusual low quality. The exclusion criterion from the CRLBs was that if absolute CRLBs of more than half of the evaluated metabolites were larger than 1.5 times the respective median value from the cohort (27), then the spectra were discarded. In addition, we excluded spectra with a Gaussian linewidth of more than 7.5 Hz in model fitting from our analysis. For absolute quantification, signal intensities were converted into millimolar (mM) concentrations based on parenchymal water derived from the TE series of unsuppressed water acquisitions in FiTAID assuming two water compartments (35). Metabolite concentrations ( ${\left[M\right]}_{\mathrm{molar}}$ ) were estimated based on the following equation (34):


$$\begin{array}{l} {\left[M\right]}_{\mathrm{molar}}=\frac{S_{M}}{S_{H_{2}O}}*\frac{1}{1-\exp\left(-\frac{T_{R}}{T_{1}}\right)}*\frac{1}{\exp\left(-\frac{T_{E}}{1.5*T_{2}}\right)}\\ \;*\left(f_{GM}d_{GM}+f_{WM}d_{WM}\right)\;{\left[H_{2}O\right]}_{\mathrm{molar}} \end{array}$$

$S_{M}$ and $S_{H_{2}O}$ indicate the metabolite and water signal intensities, as estimated in FitAID including the scaling factors for the respective number of protons per molecule. $S_{H_{2}O}$ refers to the relaxation-corrected (from TE-series) signal of parenchymal water (i.e., excluding CSF). Metabolite relaxation corrections used relaxation times from literature (36, 37), where T2 values were multiplied by the factor 1.5 to accommodate the slower T2 signal decay for sLASER (31, 38). $f_{GM}$ and $f_{WM}$ refer to the volume fractions of gray matter (GM) and white matter (WM) in the voxel, which are needed to fine-tune the water content to convert to molar concentrations. Small individual differences with even smaller effect on the water content were neglected and values derived from literature (31, 39) were used throughout. Resulting $f_{GM}$ and $f_{WM}$ amounted to 0.67 and 0.33 for hippocampus, and 0.81 and 0.19 for the reference ROI, respectively. $d_{GM}$ and $d_{WM}$ are the GM- and WM-specific water content, taken from literature [0.78 for GM, 0.65 for WM (35)]. ${\left[H_{2}O\right]}_{\mathrm{molar}}$ refers to the molar concentration of water, which is 55.01 moles/L (34).

### Voxel-based morphometry (VBM) analysis

To investigate potential volumetric changes in the right hippocampus, VBM was performed in SPM12 (University College London, London, UK) using T1-w MPRAGE images (29). First, MPRAGE images were segmented into GM, WM, and CSF applying a unified segmentation method (40). This was followed by template generation for image registration with the Diffeomorphic Anatomical Registration Through Exponentiated Lie algebra (DARTEL) algorithm (41). Based on this template, all subjects were spatially normalized to Montreal Neurological Institute (MNI) space representing the common space. Normalized images were smoothed using a Gaussian kernel with full width at half maximum of 3 mm. Next, an anatomical mask was created with the SPM anatomy toolbox (42) for the voxel-wise statistical analysis.

### Statistical analysis

Statistical analyses were performed using RStudio software (RStudio PBS, version 1.4.1106). Voxel-wise statistical analysis of the right hippocampus VBM was conducted in SPM12 (29). An unpaired two-tailed *t*-test was used to explore the difference between the mean age of SCI patients and healthy controls. To test for differences in the distribution of sex and highest graduation, the Fisher’s exact test and the chi-squared test were applied, respectively. These tests were used as they both allow comparing the frequency of a categorial variable (sex and graduation) between groups. The threshold for significant differences was set to *p* < 0.05. SCI patients were compared to healthy controls regarding differences in immediate memory performance (t1), intermediate memory performance (t2), and delta score between both time-points using unpaired two-tailed *t*-tests or Wilcoxon rank-sum tests (Mann–Whitney *U* test) in case of a non-normal distribution (tested with the Shapiro Wilk test). Potential differences in memory performance were also explored between the two educational stages groups (elementary school or secondary school vs. high school or university). These tests were furthermore used to investigate differences in metabolite concentrations between SCI patients and healthy controls for the right hippocampus and the reference region. Besides the classical *t*-test and Wilcoxon rank-sum test, Bayesian unpaired t-test and Bayesian Mann–Whitney *U* test were conducted, which allow estimating the evidence in favor of the null hypothesis. If the Bayes Factor was above 3, substantial evidence in favor of the alternative hypothesis was assumed, and a Bayes Factor below 0.33 was judged as substantial evidence in favor of the null hypothesis. Bayesian statistic was performed in the software JASP (version 0.14.1, University of Amsterdam, Amsterdam, NL). Pearson correlation was performed to investigate the correlation between memory performance and age. Correlation between hippocampal metabolite concentrations and time since injury was assessed with Pearson correlation for tNAA, mI, and tCr, and with Kendall rank correlation (for non-normal distribution) for tCho and Glx. To investigate brain volume changes at the group level, voxel-wise statistical analysis (29) was performed in the right hippocampus. In SPM12 an unpaired t-test was conducted in which total intracranial volume (TIV), age, and sex were included as covariates of no interest. Family-wise error (FWE) correction was applied, and peak-voxel threshold was set to *p* < 0.05. A post-hoc power analysis was conducted to evaluate minimal metabolite concentration alterations that would have been detected with this study population using as assumptions *n* = 26 for patients and *n* = 18 for controls, normal data distribution for both cohorts, 80% of statistical power, within group standard deviation (SD) of 10% for both groups (as for instance found for tNAA or mI).

## Results

### Demographics and clinical characteristics of study participants

This study included 28 chronic traumatic SCI patients and 18 age-, sex-, and education-matched healthy controls. Their mean age ± SD was 47.8 ± 12.8 years and 49.4 ± 12.5 years, respectively (age range of 21–70 years). There was no difference in the mean age between the two groups (*p* = 0.67). Neither sex (*p* = 0.37) nor graduation levels (*p* = 0.50) were different between SCI patients and healthy controls. The median time since injury of SCI patients was 12.5 years with a minimum of 2 years and a maximum of 37 years. Eighteen patients were tetraplegic and 10 patients were paraplegic, as defined by the International Standards for the Neurological Classification of Spinal Cord Injury (ISNCSCI) (43). Twelve patients had a sensorimotor complete lesion according to the American Spinal Injury Association (ASIA) Impairment Scale (AIS) (AIS grade A) and 16 had an incomplete (AIS grades B-D) lesion (Table 1). None of the study participants with ALFI-MMSE assessment showed signs of dementia. Only one patient was at the border of mild cognitive impairment (MCI).

### Visuospatial and verbal memory performance

**Figure 1:**
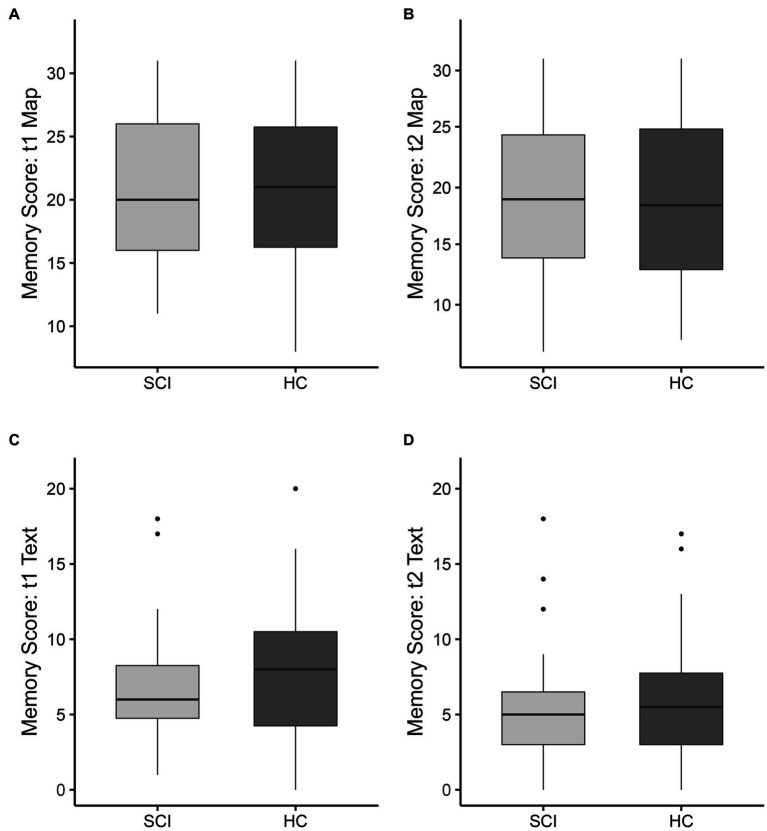
Memory scores for the visuospatial (‘Map’) memory task **(A,B)** and verbal (‘Text’) memory task **(C,D)** for spinal cord injury patients (SCI, indicated in light gray) and healthy controls (HC, indicated in dark gray). The boxplots show the median, interquartile range, and 25th and 75th percentile whiskers of memory performance for **(A,C)** immediate memory (t1) and **(B,D)** intermediate memory (t2, after ~90 min). Group differences were not significant for both tasks and both time points.

The visuospatial ‘Map’ task revealed no differences in the mean memory performance between SCI patients and healthy controls for immediate (t1) recall (21.0 ± 6.0 vs. 20.2 ± 6.8, *p* = 0.70, Figure 1A) or intermediate (t2) recall (19.4 ± 6.8 vs. 18.5 ± 8.0, *p* = 0.70, Figure 1B). Furthermore, Bayesian analysis resulted in Bayes Factors smaller than 0.33 for both time-points (BF_10_ = 0.317 at t1, BF_10_ = 0.317 at t2). There was also no difference in memory performance in the verbal ‘Text’ task between SCI patients and healthy controls at t1 (6.8 ± 4.0 vs. 8.1 ± 5.0, *p* = 0.38, Figure 1C) or t2 (5.6 ± 4.0 vs. 6.5 ± 4.8, *p* = 0.60, Figure 1D). The corresponding Bayes Factors were slightly above and below the threshold of 0.33, respectively (BF_10_ = 0.350 at t1, BF_10_ = 0.312 at t2).

**Figure 2:**
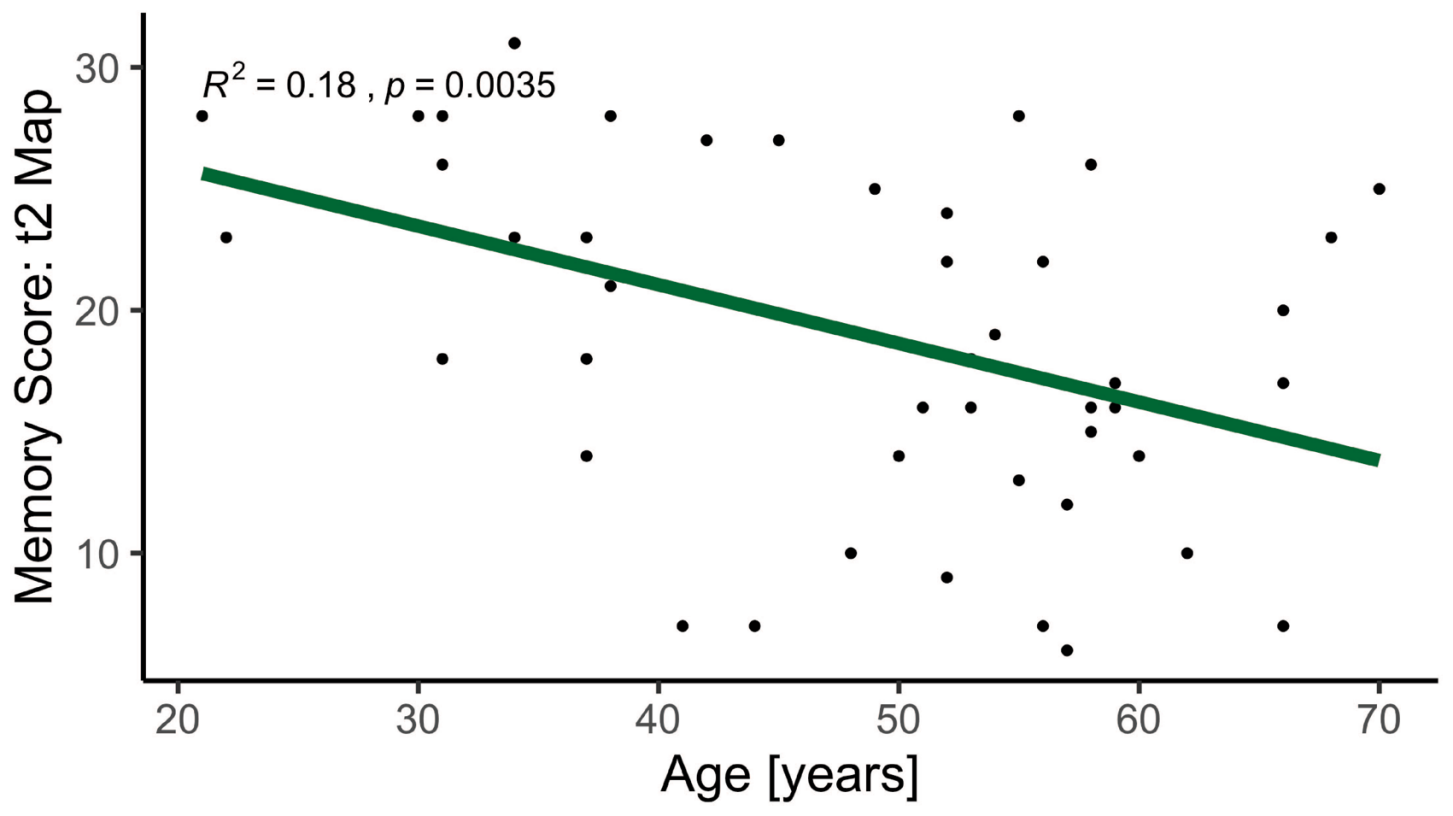
Correlation between intermediate (t2) memory score for visuospatial memory (‘Map’) and age for both spinal cord injury patients and healthy controls. Younger participants reached a higher score in the visuospatial memory test. The linearly fitted line is indicated in green.

The difference in memory performance over time, calculated as the score for intermediate (t2) recall minus the score for immediate (t1) recall, was not different for the ‘Map’ task (*p* = 0.98) or the ‘Text’ task (*p* = 0.50). Age was negatively correlated with the memory score for the immediate (t1) memory (*R*^2^ = 0.14, *p* = 0.01) and intermediate (t2) memory (*R*^2^ = 0.18, *p* < 0.01, Figure 2). There was no significant correlation between age and ‘Text’ memory score at the immediate (t1) time-point or intermediate (t2) time-point.

There was a significant difference in memory performance between the two educational stages groups (elementary school or secondary school vs. high school or university), irrespective of SCI. The group with a higher education performed significantly better on average in the ‘Map’ task at immediate (t1) recall (*p* = 0.04) and at intermediate (t2) recall (*p* = 0.01). This group also revealed higher memory performance in the ‘Text’ task at immediate (t1) recall (*p* = 0.03) and at intermediate (t2) recall (*p* = 0.04).

### Quality of magnetic resonance spectroscopy measurements

**Figure 3:**
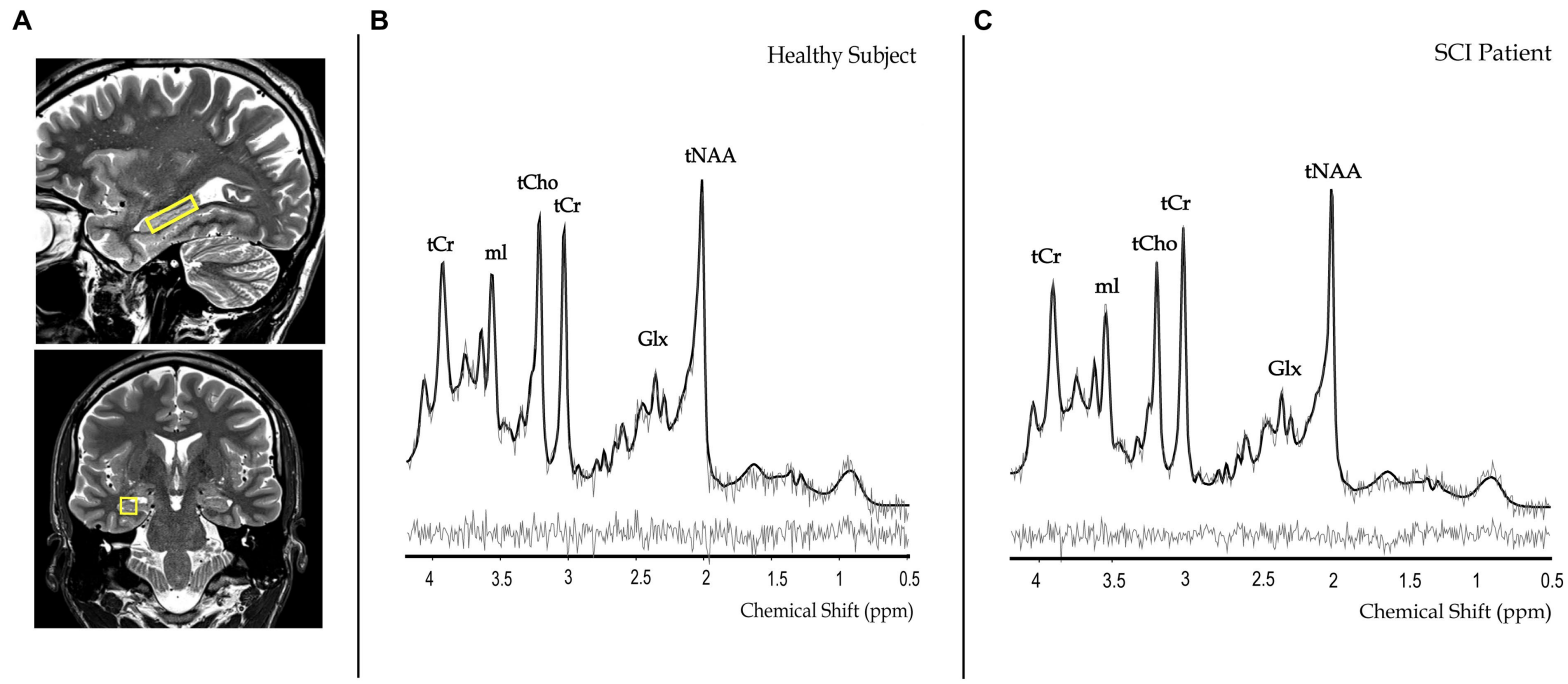
Representative metabolite spectra and planning images of spectroscopic voxel placement in the right hippocampus **(A)** of a healthy control subject **(B)** and a spinal cord injury (SCI) patient **(C)**. The voxel of interest is indicated in yellow and overlaid on sagittal and coronal T2-weighted images. Representative metabolite spectra include the fitted (black lines) and original spectra (gray lines), based on the average of 2 spectra (128 shots each). tNAA, total N-acetylaspartate; mI, myo-inositol; tCr, total creatine; tCho, choline-containing compounds; Glx, glutamate and glutamine; ppm, parts-per-million.

**Figure 4:**
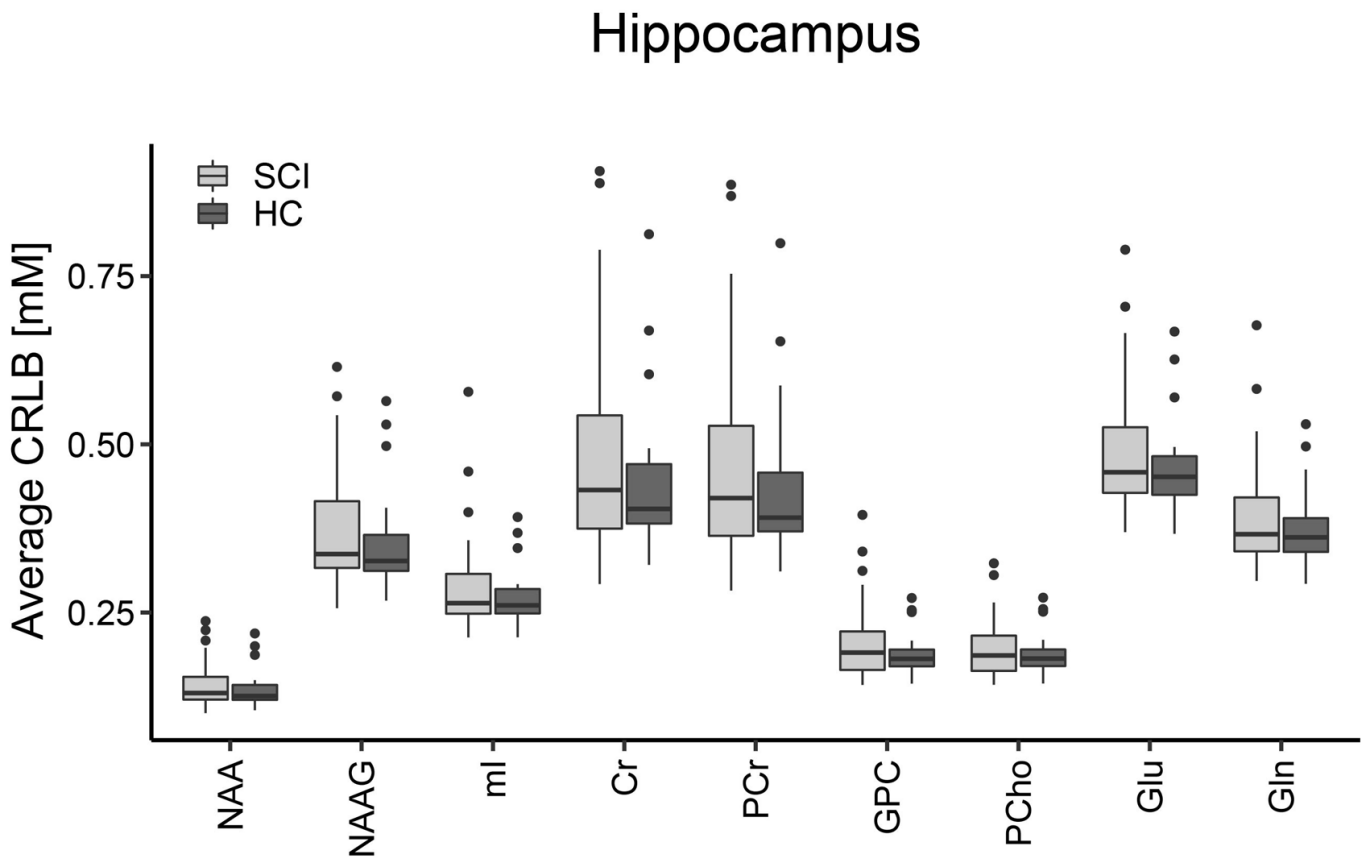
Group-averaged Cramér-Rao lower bounds (CRLBs) for the constituents of all metabolite measures investigated in the right hippocampus. The boxplots show the median and interquartile range of the average CRLB which was below 0.5 mM for all of these metabolites. Spinal cord injury patients (SCI) are indicated in light gray and healthy controls (HC) are indicated in dark gray. NAA, N-acetylaspartate; NAAG, N-acetylaspartylglutamate; mI, myo-inositol; Cr, creatine; PCr, phosphocreatine; GPC, glycerophosphorylcholine; PCho, phosphorylcholine; Glu, glutamate; Gln, glutamine.

Representative metabolite spectra acquired in the right hippocampus (Figure 3A) of a healthy control and a SCI patient are shown in Figures 3B,C, respectively. MRS data of the reference region was missing for one participant as the scan had to be interrupted. The mean Cramer-Rao lower bound (CRLB) was below 0.5 mM for all metabolites of interest in the right hippocampus, this was the case even for metabolites that were not reported individually and were instead included as parts of lumped compounds due to overlapping metabolite patterns. Due to their greater fitting uncertainties, only the total contents of correlated summed metabolites were used in the statistical analyses. Figure 4 provides a visual representation of these findings.

The CRLBs were consistently about four times lower for the reference region due to the larger VOI (hence SNR) and smaller linewidth. No significant differences were observed between the CRLBs in SCI patients and healthy controls. Based on the exclusion criteria, three subjects (two SCI patients, one healthy control) were excluded from the right hippocampus analysis and two subjects (one SCI patient, one healthy control) were excluded from the reference region analysis.

### Metabolite concentrations in the right hippocampus

The estimated concentrations and the group SD for all estimated metabolites in the hippocampus and reference region are listed in Supplementary Table 1.

**Figure 5:**
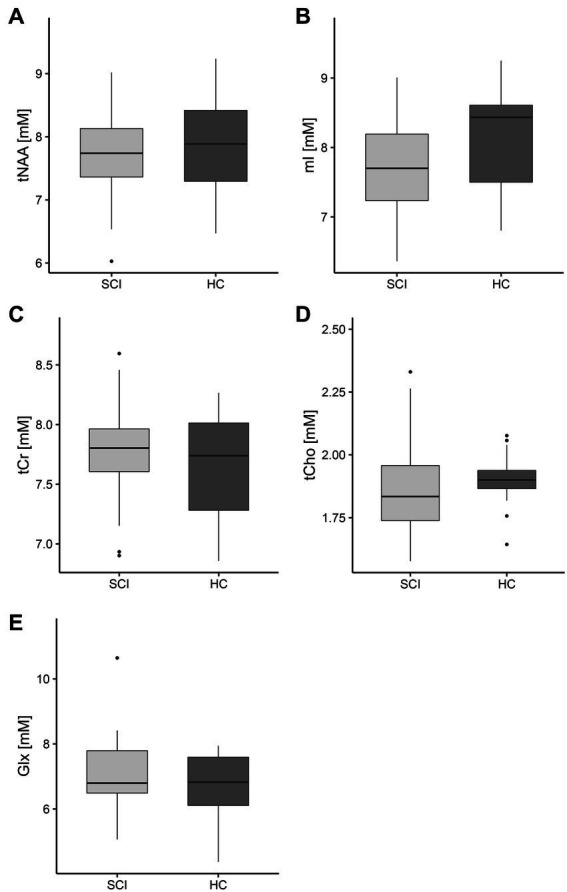
Hippocampal concentrations of all metabolites of interest. The boxplots show the median, interquartile range, and 25th and 75th percentile whiskers of **(A)** total N-acetylaspartate, **(B)** myo-inositol, **(C)** total creatine, **(D)** choline-containing compounds, and **(E)** glutamate and glutamine concentrations in the right hippocampus for spinal cord injury patients (SCI, indicated in light gray) and healthy controls (HC, indicated in dark gray). Group differences were not significant for any of the metabolites. tNAA, total N-acetylaspartate; mI, myo-inositol; tCr, total creatine; tCho, choline-containing compounds; Glx, glutamate and glutamine.

There were no significant differences between SCI patients and healthy controls in the mean concentrations of tNAA (*p* = 0.53, Figure 5A), mI (*p* = 0.09, Figure 5B), tCr (*p* = 0.59, Figure 5C), tCho (*p* = 0.27, Figure 5D), or Glx (*p* = 0.41, Figure 5E) in the right hippocampus. A sub-group analysis comparing SCI patients with a sensorimotor complete lesion (AIS grade A, *n* = 11) against healthy controls did not reveal significant differences in the mean concentration of tNAA (*p* = 0.54), mI (*p* = 0.33), tCr (*p* = 0.36), tCho (*p* = 0.61), or Glx (*p* = 0.54). Similarly, SCI patients with a cervical lesion (tetraplegics, *n* = 17) did not show significant differences with healthy controls in the mean concentration of tNAA (*p* = 0.65), mI (*p* = 0.06), tCr (*p* = 0.97), tCho (*p* = 0.24), or Glx (*p* = 0.56). Within the SCI patients’ group, no significant correlation was found between neurological level of injury (NLI) and concentrations of tNAA (*R* = −0.19, *p* = 0.19), mI (*R* = −0.05, *p* = 0.72), tCr (*R* = 0.10, *p* = 0.47), tCho (*R* = 0.04, *p* = 0.79), or Glx (*R* = 0.04, *p* = 0.79) and between time since injury and concentrations of tNAA (*R* = 0.37, *p* = 0.08), mI (*R* = 0.24, *p* = 0.26), tCr (*R* = 0.26, *p* = 0.22), tCho (*R* = 0.14, *p* = 0.33), or Glx (*R* = −0.05, *p* = 0.75).

### Metabolite concentrations in the reference region

Quantification of metabolite concentrations in the posterior parietal lobe did not reveal significant differences in tNAA (*p* = 0.97), mI (*p* = 0.06), tCr (*p* = 0.87), tCho (*p* = 0.43), or Glx (*p* = 0.56) when comparing SCI patients with healthy controls.

### Volumetric assessment of the right hippocampus

Macrostructural VBM analysis of the right hippocampus did not show significant differences in hippocampal volume between SCI patients and healthy controls.

## Discussion

This study determined the metabolic fingerprint and volumetric changes of the right hippocampus of chronic traumatic SCI patients by applying non-invasive MRS and MRI, as well as explored relationships between metabolite concentrations and cognitive deficits. Immediate and intermediate (recall ~90 min after memorizing) memory function, investigated with the VVM test, did not reveal differences between SCI patients and healthy controls for both visuospatial and verbal memory performance. In line with normal cognitive function, the right hippocampus remained unaffected in chronic SCI on a molecular and structural level compared with age-, sex-, and education-matched healthy controls. The absence of MRS-derived metabolic changes and structural MRI-derived atrophy suggests that there is no ongoing neurodegeneration evident in the right hippocampus in chronic SCI patients.

While a few studies previously found cognitive deficits in patients with SCI, such as learning and memory impairments (2–5, 15), we did not detect differences in immediate or intermediate visuospatial or verbal memory performance between SCI patients and healthy controls. This may be attributable to varying study designs and cohorts, different cognitive tests assessed, varying educational levels, confounding factors such as psychological or cognitive comorbidities, and concomitant traumatic brain injury (TBI) (4, 5, 44–46). Variables that might be indirectly related to memory performance following SCI, such as age, sex, and education, were matched to healthy controls in this study. These factors, however, resulted in a considerable variation of memory scores in both groups which seems to be expected as other studies conducting the VVM reported similar degrees of variation (20, 22). We also had strict inclusion criteria to exclude participants with severe TBI and related cognitive impairments, neurological or mental diseases, alcohol or substance abuse, as well as major depression or anxiety (3–5, 7, 44, 46). Especially TBI has been linked to neuroinflammation and impacted spatial (47) and verbal (48) memory function. While TBI patients were reported to have an increased risk to develop dementia (49), elevated risks of dementia in SCI patients became non-significant when correcting for co-morbid factors (50). We specifically tested for signs of dementia in our study participants by conducting the telephone version of the MMSE and were able to control for this measurement confound by showing that all participants were in the normal cognitive range besides one who was at the border of being mildly demented.

In line with the functional data on visuospatial and verbal memory performance, tNAA, mI, tCr, tCho, and Glx metabolites did not change significantly in chronic SCI patients in this study. Crucially, no differences were detected despite applying an advanced MRS technique, optimized for the case of low SNR data from small regions of interest in the brain (27, 31, 32). The quality of the recorded MR spectra is excellent when comparing to the previous best-practice reports for the hippocampus (31, 32), both by visual judgment of the spectral quality and with regard to the achieved spectral resolution. The CRLBs, a basic measure for measurement precision, are equivalent to those achieved previously with much longer measurements [256 acquisitions here vs. 768–2,304 acquisitions (31)] or with a more generously prescribed hippocampal ROI (50% larger ROI) in (32). Similarly, the confidence limits for the cohort mean concentration values are tighter for almost all metabolites than those reported before in (31) for 3.6 times longer acquisitions (and even when measurements of 3 repeated sessions were combined). In comparison to (32) the limits are similar with metabolite-specific differences in one or the other direction. We were able to define the normal hippocampal range of metabolite content for major metabolites with a group standard deviation of ~10% using the MC method and optimized post-processing. This would allow to detect fairly small (<10%) cohort differences for normally distributed values, given the currently investigated number of subjects.

The missing disease effect is in contrast to AD patients exhibiting decreased levels of the neuronal marker tNAA and excitatory neurotransmitter glutamate (main constituent of Glx) as well as increased levels of the gliosis marker mI in the hippocampus (51, 52), indicative of chronic neuroinflammation and neurodegeneration (8). Moreover, recent studies identified a distinct neuroinflammatory and neurodegenerative metabolic profile in the supralesional cervical spinal cord (53, 54) and cortical regions (55) in chronic SCI, characterized by changes in tNAA, mI, and tCho levels. In line with the functional findings and the neurochemical profile, the macrostructural assessment of the right hippocampus using VBM did not reveal any volumetric change in SCI patients when compared to healthy controls. This indicates there may not be hippocampal atrophy in chronic SCI, in contrast to other human neurological disorders, such as AD (9, 51), and preclinical SCI studies demonstrating ongoing neuroinflammation and -degeneration as well as impaired neurogenesis (13, 14, 56).

This discrepancy between experimental animal models and findings from patients may partially be explained by different methodological approaches and the controlled laboratory setting (1). Human SCI cohorts, by nature present rather heterogeneous demographical, etiological, and clinical characteristics as well as different genetics and pathophysiological processes. Specifically, SCI patients in our study showed a large variability in NLI (from C2 to T12), AIS grade (AIS grade A to D), age (22–68 years old), and time since injury (2–37 years). While lower lesion levels and less severe injuries were shown to be related to less structural and molecular neurodegenerative changes remote from the lesion (54, 57) as well as smaller cognitive deficits (45), a younger age has also been reported to be linked to better cognitive functioning (4) and a higher potential of regenerative axonal sprouting and repair after central nervous system damage (58). Additionally, cognitive performance was shown to decrease with increasing time since SCI (2).

Although previous studies reported several factors as potential contributors to cognitive deficits following SCI, including functional MRI (fMRI) correlates of motivational and affective sequelae (59), none of them was able to identify potential pathological mechanisms or neural substrates underlying memory impairments. The absence of direct SCI-related pathology within the hippocampus may be explained by the fact that there are no ascending or descending tracts directly connecting the spinal cord and the hippocampus (60) and thus no primary anterograde or retrograde degeneration of axons projecting to the hippocampus. Indeed, to the best of our knowledge, no studies have so far explored SCI-induced metabolic or structural changes in the hippocampus by means of MRS or MRI. If there were in fact injury-induced molecular and/or structural changes at the level of the hippocampus, especially at the early stages following SCI, it may be due to normalizing over time for endogenous neuroprotective and -regenerative processes or reorganization and compensation induced by exogeneous factors such as rehabilitation and improved visuospatial skills, e.g., due to wheelchair usage (12, 58, 61). Importantly, this study does not disprove previous findings of cognitive decline after SCI, but rather provides complementary information about the factors potentially underlying cognitive deficits following SCI. Based on our results consistently found over all outcome measures, it seems that there is no molecular or structural hippocampus pathology that may drive cognitive decline after SCI and that visuospatial and verbal memory are not significantly affected.

It is necessary to consider limitations of this study. Detection of functional cognitive deficits in our study was limited to the tasks implemented in the VVM test which examine hippocampus-dependent visuospatial and verbal memory. These are restricted to learning and memory and might not be sensitive to detect impairments in other cognitive domains. Moreover, the VVM test has previously demonstrated a higher level of SD when it included participants from a wide age range and diverse educational backgrounds (18), as compared to the current study. In contrast, the present study also included both MRS and MRI measures to quantitatively investigate the effects of SCI on cognitive function. However, complementary assessment of the MMSE (23), which is used in clinical settings and research to measure cognitive impairment and for AD diagnosis, was applied in both groups. MMSE results were in line with VVM test, did not show cognitive decline, and helped to exclude AD subjects. Another consideration is the coverage of different hippocampal subregions by the VOI used for metabolic analysis. The possibility of smaller voxels focusing on specific subregions would be interesting as preclinical studies have shown injury-induced secondary neuron loss in particular parts of the hippocampus (56). However, to achieve a satisfying signal-to-noise ratio in MRS measurements, a minimal VOI size is necessary (62). Furthermore, the detection of group effects is limited by the cross-sectional study design. Tracking molecular changes over time may be more sensitive as it minimizes the effect of variation among individuals. A longitudinal study design can allow both detecting potential biochemical differences within participants and groups and monitoring the evolution of atrophic changes and cognitive performances over time to explore how these relate to the dynamics of the hippocampal metabolic profile. The study has a disproportionate number of female participants. If we were to investigate whether gender has an impact on metabolic concentrations or memory function vulnerability, a much larger sample size would be necessary. For this study, we aimed to minimize bias by ensuring an equal distribution of gender across all groups.

This study suggests that the right hippocampus may not be pathologically affected at a functional, metabolic, and volumetric level in chronic SCI by means of non-invasive multimodal neuroimaging. While previous findings of cognitive decline after SCI can be attributable to methodological study differences, secondary consequences of SCI, or changes in lifestyle, the absence of trauma-induced metabolic and structural changes as well as memory impairments in this study speaks against secondary neurodegeneration in the right hippocampus years after SCI. Longitudinal studies may improve our understanding of the dynamic molecular and structural processes along the neuraxis and if/how these relate to cognitive functioning following SCI.

## Data availability statement

The original contributions presented in the study are included in the article/Supplementary material, further inquiries can be directed to the corresponding author.

## Ethics statement

The studies involving human participants were reviewed and approved by the local Ethics Committee Cantonal Zurich (EK-2018-00937). The patients/participants provided their written informed consent to participate in this study.

## Author contributions

DP: study concept and design, acquiring data, data analysis, interpretation of data, and writing the manuscript. SZ: study design, data analysis, acquiring data, interpretation of data, and critical revision of manuscript for intellectual content. KŞ: data analysis and critical revision of manuscript for intellectual content. RK: study concept and design, acquiring data, data analysis, interpretation of data, and critical revision of manuscript for intellectual content. PF: study concept and design, critical revision of manuscript for intellectual content, and writing the manuscript. MS: study concept and design, acquiring data, data analysis, interpretation of the data, writing the manuscript, and critical revision of manuscript for intellectual content.

## Funding

MS received grants from Wings For Life charity (No. WFL-CH-19/20), grants from International Foundation for Research (IRP-158), and PF received a personal grant from the Swiss National Science Foundation (SNF, No. 181362). RK and KŞ were supported by a project grant from SNF (No. 320030-175984).

## Conflict of interest

The authors declare that the research was conducted in the absence of any commercial or financial relationships that could be construed as a potential conflict of interest.

## Publisher’s note

All claims expressed in this article are solely those of the authors and do not necessarily represent those of their affiliated organizations, or those of the publisher, the editors and the reviewers. Any product that may be evaluated in this article, or claim that may be made by its manufacturer, is not guaranteed or endorsed by the publisher.

## Glossary

**Table:** 

AIS	American Spinal Injury Association Impairment Scale
Cho	choline
Cr	creatine
CRLB	Cramer-Rao lower bounds
CSF	cerebrospinal fluid
FWE	family-wise error
FitAID	fitting tool for arrays of interrelated datasets
GABA	γ-aminobutyric acid
Gln	glutamine
Glu	glutamate
Glx	glutamate plus glutamine
GM	gray matter
GPC	glycerophosphorylcholine
sLASER	semi localization by adiabatic selective refocusing
MC	metabolite cycling
mI	myo-inositol
MMSE	Mini-Mental State Examination
MPRAGE	magnetization-prepared rapid acquisition with gradient echo
MRI	magnetic resonance imaging
MRS	magnetic resonance spectroscopy
MS	multiple sclerosis
NAA	N-acetylaspartate
NAAG	N-acetylaspartylglutamate
NLI	neurological level of injury
PCho	phosphorylcholine
PCr	phosphocreatine
ppm	parts-per-million
ROI	region of interest
SCI	spinal cord injury
TBI: traumatic brain injury	
tCho	choline-containing compounds (PCho plus GPC)
tCr	total creatine (Cr plus PCr)
TIV	total intracranial volume
tNAA	total N-acetylaspartate (NAA plus NAAG)
VBM	voxel-based morphometry
VVM	Visueller und verbaler Merkfähigkeitstest
WM	white matter

## Glossary

AIS: American Spinal Injury Association Impairment Scale
Cho: choline
Cr: creatine
CRLB: Cramer-Rao lower bounds
CSF: cerebrospinal fluid
FWE: family-wise error
FitAID: fitting tool for arrays of interrelated datasets
GABA: γ-aminobutyric acid
Gln: glutamine
Glu: glutamate
Glx: glutamate plus glutamine
GM: gray matter
GPC: glycerophosphorylcholine
sLASER: semi localization by adiabatic selective refocusing
MC: metabolite cycling
mI: myo-inositol
MMSE: Mini-Mental State Examination
MPRAGE: magnetization-prepared rapid acquisition with gradient echo
MRI: magnetic resonance imaging
MRS: magnetic resonance spectroscopy
MS: multiple sclerosis
NAA: N-acetylaspartate
NAAG: N-acetylaspartylglutamate
NLI: neurological level of injury
PCho: phosphorylcholine
PCr: phosphocreatine
ppm: parts-per-million
ROI: region of interest
SCI: spinal cord injury
TBI: traumatic brain injury
tCho: choline-containing compounds (PCho plus GPC)
tCr: total creatine (Cr plus PCr)
TIV: total intracranial volume
tNAA: total N-acetylaspartate (NAA plus NAAG)
VBM: voxel-based morphometry
VVM: Visueller und verbaler Merkfähigkeitstest
WM: white matter
