# Supplementary material for: Magnetic resonance spectroscopy investigation in the right human hippocampus following spinal cord injury
Source: Front Neurol. 2023 May 12;14:1120227. doi: 10.3389/fneur.2023.1120227 (PMC10213741; doi:10.3389/fneur.2023.1120227)
Supplement: Supplementary file 1 [file Data_Sheet_1.docx]

###
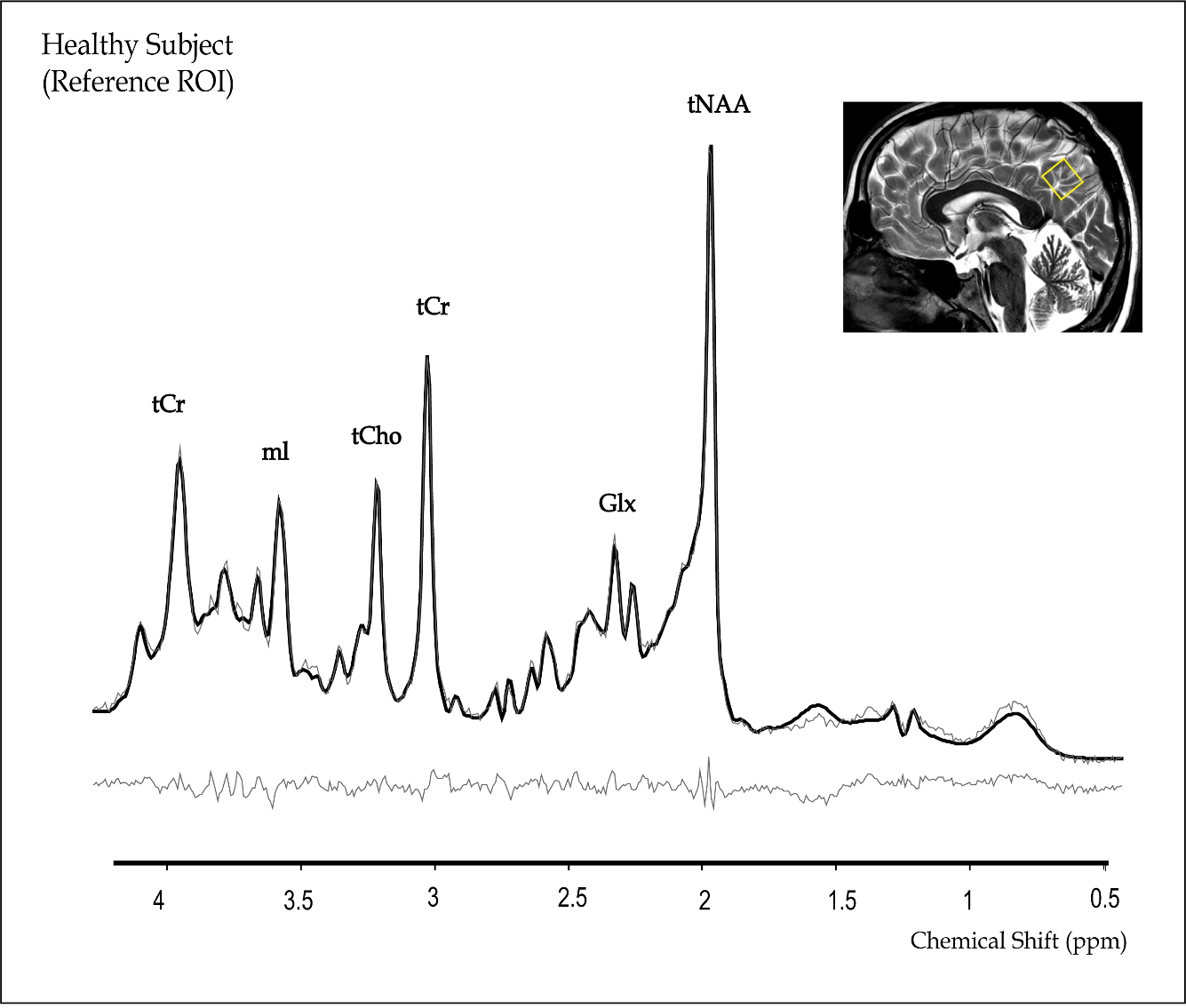


### ***Supplementary figure 1****: Representative metabolite spectra and planning images of spectroscopic voxel placement the reference region within the posterior parietal lobe of a healthy control. Representative metabolite spectra include the fitted (black lines) and original spectra (grey lines), based on the average of 2 spectra (128 shots each). tNAA = total N-acetylaspartate; mI = myo-inositol; tCr = total creatine; tCho = choline-containing compounds; Glx = glutamate and glutamine; ppm = parts-per-million.*


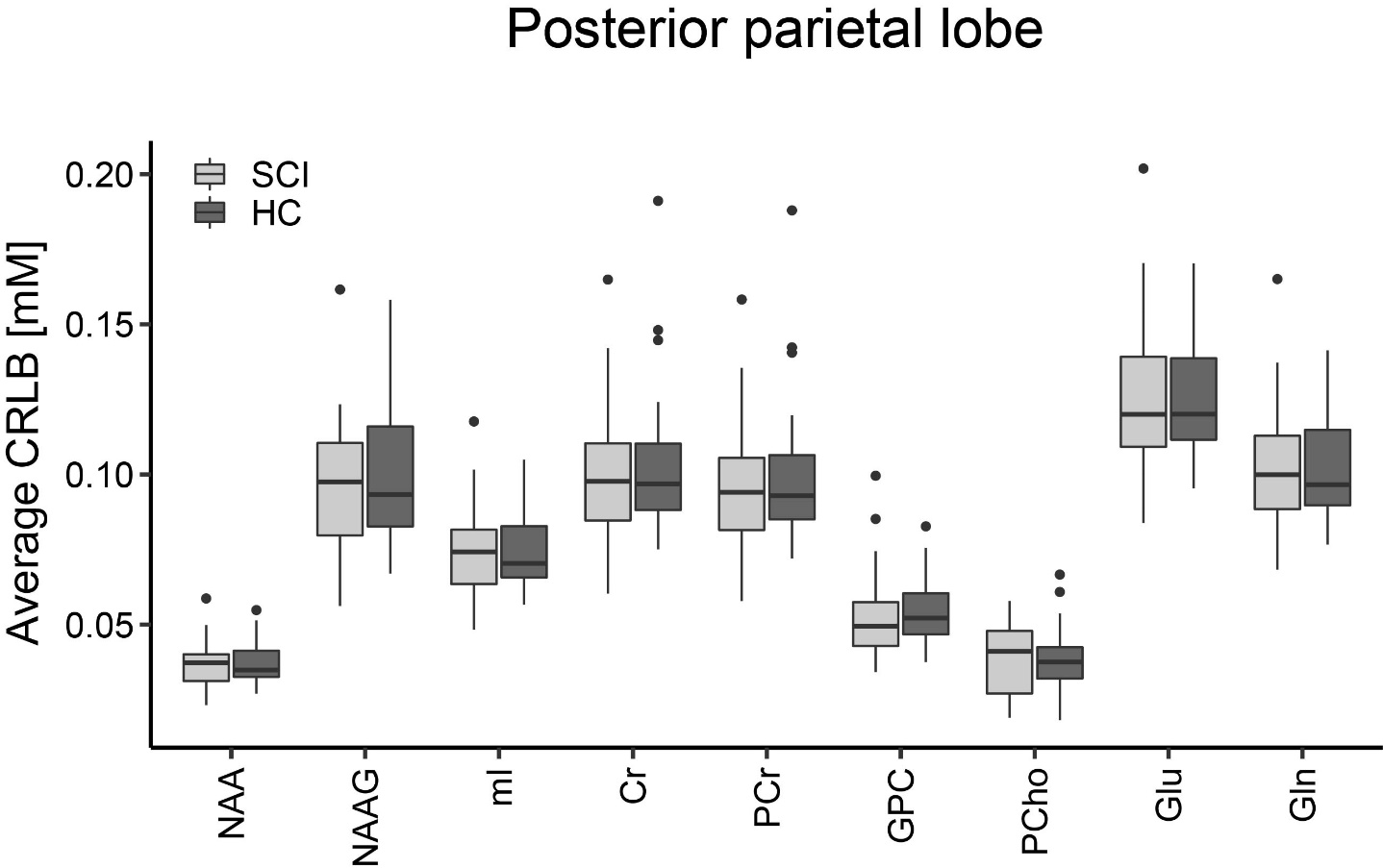
***Supplementary figure 2****: Average Cramér-Rao lower bounds (CRLBs) for all metabolites investigated in the reference region within the posterior parietal lobe. The boxplots show the median, interquartile range, and 25th and 75th percentile whiskers of the average CRLB which was below 50% for all metabolites of interest. Spinal cord injury (SCI) patients are indicated in light grey and healthy controls (HC) are indicated in dark grey. NAA = N-acetylaspartate; NAAG = N-acetylaspartylglutamate; mI = myo-inositol; Cr = creatin; PCr = phosphocreatine; GPC = glycerophosphorylcholine; PCho = phosphorylcholine; Glu = glutamate; Gln = glutamine.*


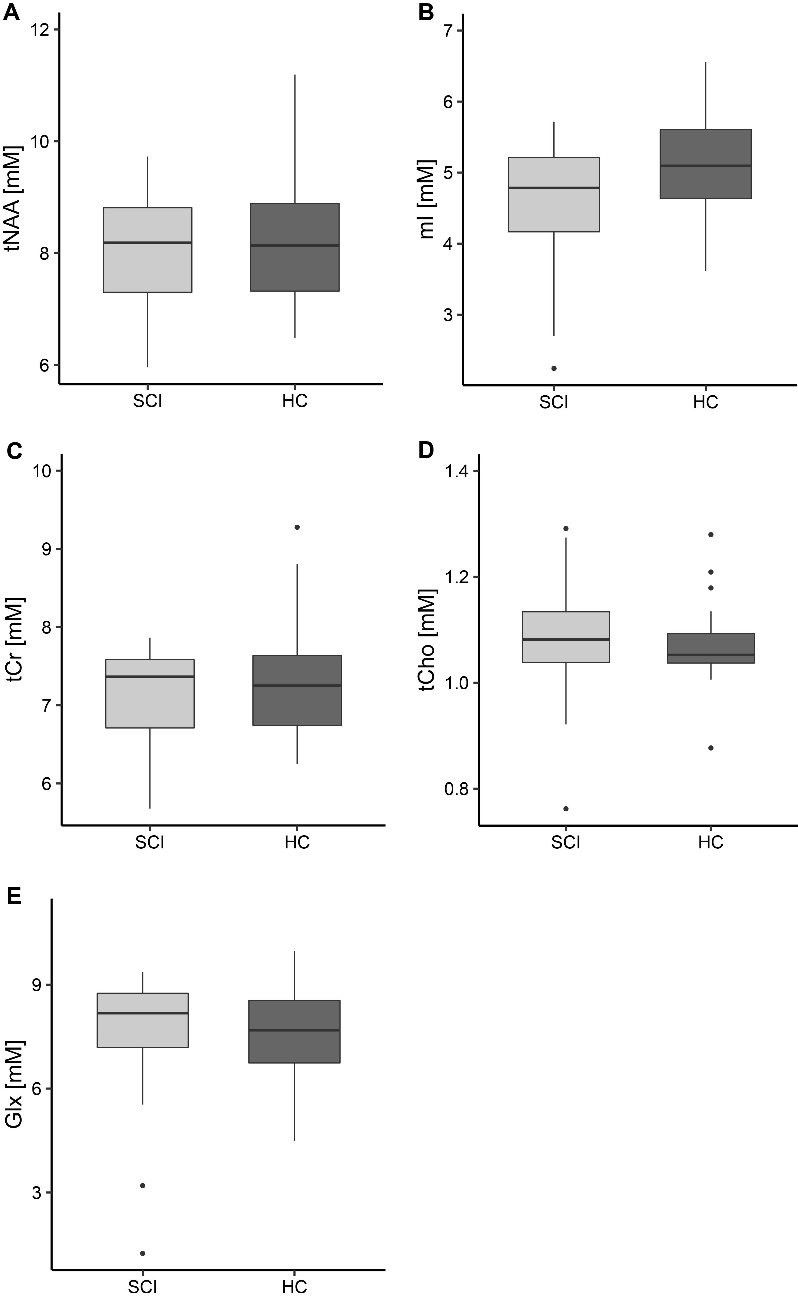


***Supplementary figure 3****: Concentrations of all metabolites of interest in the posterior parietal lobe. The boxplots show the median, interquartile range, and 25^th^ and 75^th^ percentile whiskers of metabolite concentrations in the reference region within the posterior parietal lobe for spinal cord injury patients (SCI, indicated in light grey) and healthy controls (HC, indicated in dark grey). Group differences were not significant for any of the metabolites. tNAA = total N-acetylaspartate; mI = myo-inositol; tCr = total creatine; tCho = choline-containing compounds; Glx = glutamate and glutamine.*

| Metabolic concentration [mM] | Hippocampus | | | | Posterior parietal lobe | | | |
| --- | --- | --- | --- | --- | --- | --- | --- | --- |
|  | SCI | | HC | | SCI | | HC | |
|  | Mean | SD | Mean | SD | Mean | SD | Mean | HD |
| tCr | 7.78 | 0.45 | 7.71 | 0.44 | 7.14 | 0.59 | 7.33 | 0.83 |
| tNAA | 7.74 | 0.69 | 7.89 | 0.84 | 8.04 | 1.04 | 8.24 | 1.37 |
| tCho | 1.87 | 0.19 | 1.90 | 0.11 | 1.09 | 0.11 | 1.07 | 0.09 |
| Glx | 7.10 | 1.11 | 6.68 | 1.06 | 7.58 | 1.91 | 7.57 | 1.45 |
| Glucose | 1.15 | 0.67 | 1.35 | 0.61 | 0.74 | 0.55 | 0.87 | 0.63 |
| Aspartate | 1.99 | 0.78 | 2.05 | 0.57 | 2.74 | 0.92 | 2.94 | 0.78 |
| Creatine | 3.39 | 0.76 | 3.03 | 0.71 | 4.40 | 0.50 | 4.60 | 0.57 |
| GABA | 2.01 | 1.07 | 1.79 | 0.69 | 2.73 | 0.91 | 2.58 | 0.30 |
| Glutamate | 4.96 | 0.77 | 4.77 | 0.79 | 5.50 | 1.64 | 5.68 | 1.10 |
| Glutamine | 2.14 | 0.71 | 1.91 | 0.57 | 2.08 | 0.41 | 1.88 | 0.48 |
| Glutathione | 1.28 | 0.23 | 1.19 | 0.21 | 1.08 | 0.32 | 1.11 | 0.17 |
| Glycine | 0.48 | 0.40 | 0.47 | 0.52 | 0.96 | 0.33 | 0.87 | 0.28 |
| glycerophosphorylcholine | 0.73 | 0.34 | 0.89 | 0.36 | 0.10 | 0.11 | 0.07 | 0.10 |
| Lactate | 0.52 | 0.29 | 0.69 | 0.66 | 0.98 | 0.29 | 1.29 | 0.43 |
| Macromolecular background | 0.13 | 0.01 | 0.14 | 0.01 | 0.14 | 0.03 | 0.14 | 0.02 |
| myo-Inositol (mI) | 7.71 | 0.68 | 8.10 | 0.76 | 4.59 | 0.85 | 5.10 | 0.70 |
| N-acetylaspartate (NAA) | 5.88 | 0.37 | 5.95 | 0.42 | 7.29 | 0.58 | 7.79 | 1.04 |
| N-acetylaspartylglutamate | 1.86 | 0.60 | 1.93 | 1.05 | 0.75 | 0.62 | 0.45 | 0.43 |
| Phosphocreatine | 4.39 | 0.73 | 4.67 | 0.53 | 2.74 | 0.38 | 2.73 | 0.50 |
| Phosphorylcholine | 1.15 | 0.28 | 1.00 | 0.30 | 0.99 | 0.12 | 1.01 | 0.12 |
| Phosphorylethanolamine | 3.52 | 1.11 | 3.51 | 0.93 | 2.83 | 1.06 | 3.12 | 0.64 |
| scyllo-Inositol | 0.39 | 0.20 | 0.48 | 0.22 | 0.29 | 0.14 | 0.34 | 0.15 |
| Taurine | 1.23 | 0.61 | 1.31 | 0.56 | 0.81 | 0.32 | 0.85 | 0.48 |
| mI/tNAA | 1.00 | 0.12 | 1.04 | 0.16 | 0.57 | 0.10 | 0.62 | 0.06 |

***Supplementary table 1****: Mean concentration and standard deviation of all metabolites quantified in spinal cord injury patients (SCI) and healthy controls (HC). In addition, the metabolite ratio for the concentrations of mI and NAA, presumably the most sensitive marker for neurodegeneration, usually characterized by NAA decrease and mI increase.*

| Site (Name or Number) |  |  |
| --- | --- | --- |
| 1. Hardware | ROI (Hippocampus) | Reference ROI |
| a. Field strength [T] | 3T | 3T |
| b. Manufacturer | Siemens | Siemens |
| c. Model (software version if available) | Prisma (VE11C) | Prisma (VE11C) |
| d. RF coils: nuclei (transmit/ receive), number of channels, type, body part | 64-channel receive head and neck RF coil | 64-channel receive head and neck RF coil |
| e. Additional hardware | - | - |
| 2. Acquisition |  |  |
| a. Pulse sequence | semiLaser (home-written) | semiLaser (home-written) |
| b. Volume of Interest (VOI) locations | body and tail of right hippocampus | posterior parietal lobe |
| c. Nominal VOI size [cm^3^, mm^3^] | 2.4 cm^3^ | 6.2 cm^3^ |
| d. Repetition Time (TR), Echo Time (TE) [ms, s] | TE 35 ms, TR 2.5 s | TE 35 ms, TR 2.5 s |
| e. Total number of Excitations or acquisitions per spectrum  In time series for kinetic studies   1. Number of Averaged spectra (NA) per time-point 2. Averaging method (e.g. block-wise or moving average) 3. Total number of spectra (acquired / in time-series) | 2 times 128 | 2 times 128 |
| f. Additional sequence parameters  (spectral width in Hz, number of spectral points, frequency offsets)  If STEAM:, Mixing Time (TM)  If MRSI: 2D or 3D, FOV in all directions, matrix size, acceleration factors, sampling method | SW 4000 Hz, 4096 points, (only 2048 points [0.512 s FID length] used), excitation centered at 2.5 ppm;  For quantification, non-water-suppressed spectra with TE = 35, 1000, 50, 400, 200, 75, 100, and 140 ms, TR = 6000 ms to separate parenchymal water from CSF | SW 4000 Hz, 4096 points, (only 2048 points [0.512 s FID length] used), excitation centered at 2.5 ppm;  For quantification, non-water-suppressed spectra with TE = 35, 1000, 50, 400, 200, 75, 100, and 140 ms, TR = 6000 ms to separate parenchymal water from CSF |
| g. Water Suppression Method | Metabolite cycling | Metabolite cycling |
| h. Shimming Method, reference peak, and thresholds for “acceptance of shim” chosen | FASTESTMAP, accept for water linewidth (Gauss part of Voigt line) < 7.5 Hz | FASTESTMAP, accept for water linewidth (Gauss part of Voigt line) < 7.5 Hz |
| i. Triggering or motion correction method  (respiratory, peripheral, cardiac triggering, incl. device used and delays) | - | - |
| 3. Data analysis methods and outputs | ROI (Hippocampus) | Reference ROI |
| a. Analysis software | FiTAID | FiTAID |
| b. Processing steps deviating from quoted reference or product | Frequency and phase alignment of single acquisitions | Frequency and phase alignment of single acquisitions |
| c. Output measure  (e.g. absolute concentration, institutional units, ratio)Processing steps deviating from quoted reference or product | concentrations (mM) | concentrations (mM) |
| d. Quantification references and assumptions, fitting model assumptions | Concentrations derived from water signal (TE Loop) and assumed water content of parenchymal water and assumed relaxation times. Fit model using prior knowledge (Voigt lineshape, common Lorentz and Gauss width) macromolecular spectrum derived from subset of data | Concentrations derived from water signal (TE Loop) and assumed water content of parenchymal water and assumed relaxation times. Fit model using prior knowledge (Voigt lineshape, common Lorentz and Gauss width) macromolecular spectrum derived from subset of data |
| 4. Data Quality | ROI (Hippocampus) | Reference ROI |
| a. Reported variables  (SNR, Linewidth (with reference peaks)) | *Gauss linewidth for metabolite peaks* | *Gauss linewidth for metabolite peaks* |
| b. Data exclusion criteria | *Majority of CRLB> 1.5 times median value over cohort, or Gauss width >7.5Hz* | *Majority of CRLB> 1.5 times median value over cohort, or Gauss width >7.5Hz* |
| c. Quality measures of postprocessing Model fitting (e.g. CRLB, goodness of fit, SD of residual) | *CRLB [mM]* | *CRLB [mM]* |
| d. Sample Spectrum | yes | yes |

***Supplementary table 2****: In vivo magnetic resonance spectroscopy checklist as suggested by recent consensus effort for reproducible research^54^ and specific details used in this study.*

-------------------------------------------------------------

* Lin A, Andronesi O, Bogner W, Choi IY, Coello E, Cudalbu C, Juchem C, Kemp GJ, Kreis R, Krssak M, Lee P, Maudsley AA, Meyerspeer M, Mlynarik V, Near J, Oz G, Peek AL, Puts NA, Ratai EM, Tkac I, Mullins PG, Experts' Working Group on Reporting Standards for MRS. Minimum Reporting Standards for in vivo Magnetic Resonance Spectroscopy (MRSinMRS): Experts' consensus recommendations. NMR Biomed 2021;34(5):e4484.
